# Risk of Guillain-Barré Syndrome Following Laboratory-Confirmed Dengue Infection

**DOI:** 10.1056/NEJMc2519008

**Published:** 2026-04-16

**Authors:** Thiago Cerqueira-Silva, Enny Paixão, Giovanny V.A. França, Luiza X.S. Tenório, Mateus C. Amaral, Nubia M. Santos, Neil Pearce, Viviane S. Boaventura, Manoel Barral-Netto

**Affiliations:** 1 London School of Hygiene & Tropical Medicine, London; 2 Ministério da Saúde, Brasília, Brazil; 3 Fundação Oswaldo Cruz, Salvador, Brazil

**To the Editor**

Dengue has spread faster than any other mosquito-borne illness, with 14 million cases reported in 2024 worldwide. Dengue can rarely cause neurological complications, including Guillain-Barré syndrome (GBS).^1^ Multiple case reports have suggested an association between GBS and dengue, but this risk has not been quantified in a large-scale study.^2^

We analysed linked national databases covering hospitalisation, dengue notification, and mortality in Brazil between 2023 and 2024. We conducted a self-controlled case series (SCCS) to estimate the incidence rate ratios (IRR) of GBS 1 to 42 days following laboratory-confirmed dengue infection identified through RT-PCR, NS1 antigen, or IgM testing compared to the control period. We used the extended SCCS model for event-dependent exposure. We conducted sensitivity analysis and positive and negative control outcomes to assess the robustness of our findings. Details regarding the data sources and statistical analysis plan are provided in the Supplementary Appendix.

We identified 5,055 GBS hospitalizations. Of these, 147 had a documented dengue infection, with 89 occurring within the risk window. The IRR during days 1–42 was 16.75 (95% CI: 10.97–25.55; p-value <0.001). Risk was highest in the first two weeks and returned to baseline levels by day 43. The attributable risk between days 1-42 was 35.5 (34.3-36.3) excess GBS cases per million laboratory-confirmed dengue infections.

Our sensitivity analyses, restricted to RT-PCR/NS1-confirmed cases and including clinically diagnosed dengue, showed consistent findings. (Table 1) Positive control outcomes (stroke and acute myocardial infarction) showed expected associations, while negative controls (foot and shoulder fractures) showed no association, supporting internal validity. (Supplementary Appendix)

The risk of GBS following dengue infection is similar to other established GBS triggers, such as influenza (IRR 16) and *Campylobacter jejuni* (OR 3–41).^3^ While *Campylobacter jejuni* and enteroviruses are considered the most common GBS triggers globally, studies from tropical regions, including Brazil, Malaysia, and India, suggest that arboviral infections may be more common triggers in these settings.^4,5^ Given the recent large-scale outbreaks of dengue in endemic regions, dengue-related GBS may represent a substantial and under-recognized component of the neurological burden attributable to dengue.

These findings have important clinical implications. Clinicians in dengue-endemic areas should suspect GBS in patients presenting with progressive weakness during or shortly after dengue infection. Recognizing symptoms early allows for timely immunotherapy (intravenous immunoglobulin or plasmapheresis), which halts disease progression and improves recovery. From a public health perspective, our results further strengthen the case for dengue vaccination programs.

## Supplementary Material

Supplementary Appendix

## Figures and Tables

**Table 1:** Incidence Rate Ratios after Laboratory-Confirmed Dengue Infection in the risk period of 1 to 42 days post symptom onset, and week-stratified.

Analysis	Number of events (risk-period)	Incidence rate ratio (95% CI) *	Attributable risk per million of infections (95% CI)	P value‡
**Main analysis**				
**Days 1 -42**	89	16.75 (10.97 to 25.55)	35.5 (34.3 to 36.3)	<0.001
**Week stratified analysis**				
Days 1-7	27	30.26 (17.05 to 53.72)	11.1 (10.8 to 11.2)	
Days 8-14	29	32.97 (19.00 to 57.20)	11.9 (11.6 to 12.1)	
Days 15-28	29	16.82 (9.86 to 28.70)	11.6 (11.0 to 11.9)	
Days 29-42	4	2.35 (0.82 to 6.75)	1.0 (-0.4 to 1.4)	
Days 43-84	5	0.99 (0.38 to 2.62)	0.0 (-3.5 to 1.3)	
**Sensitivity Analysis** **(Days 1-42)**				
NS1/RT-PCR confirmed	54	11.92 (7.33 to 19.38)	24.8 (23.4 to 25.6)	
Laboratory and clinical confirmed cases	248	17.71 (13.55 to 23.14)	37.1 (36.4 to 37.6)	

* The statistical analysis plan did not specify multiplicity adjustment for secondary analyses. Results are presented as point estimates with 95% confidence intervals, which have not been adjusted for multiplicity and should not be used in place of hypothesis testing.

† The P value was calculated from a two-sided Wald test.
