# Supplementary material for: Risk of Guillain-Barré Syndrome Following Laboratory-Confirmed Dengue Infection
Source: N Engl J Med. Author manuscript; Available in PMC 2026 Jun 15. (PMC7619180; doi:10.1056/NEJMc2519008)
Supplement: Supplementary Appendix [file EMS212719-supplement-Supplementary_Appendix.pdf]

Table of Contents

Supplementary Methods 2

Supplementary Results 5

Supplementary Discussion 6

Supplementary Figures 9

Figure S1: Illustration of the risk period for the extended and standard self-controlled case cases 10

Figure S2: Selection of study participants. 10

Figure S3: Number of Guillain-Barré syndrome (GBS) hospitalisations and laboratory-confirmed dengue cases (in thousands) by month during the study period. 11

Supplementary Tables 11

Table S1: Baseline Characteristics of Patients Who had a GBS hospitalisation within the study Period. 11

Table S2. Baseline characteristics of patients who tested positive for dengue and who had an outcome of interest within the study period. Only for Guillain-Barré syndrome (GBS) clinical cases are also included. 13

Table S3. Incidence Rate Ratios after Laboratory Confirmed Dengue Infection in the risk period of 1 to 42 days post symptom onset in the positive and negative control outcomes. 14

Table S4. Incidence Rate Ratios (IRR) of Stroke and Acute Myocardial Infarction (AMI) following Laboratory Confirmed Dengue Infection in the risk period of 1 to 84 days post symptom onset, stratified by time. 14

Supplementary References: 15

Supplementary Methods

Data sources and study period

We used data from three databases: the Hospital Information System (*Sistema de Informações Hospitalares do SUS*, SIH-SUS); Notifiable Diseases Information System (*Sistema de Informação de Agravos de Notificação*; SINAN); and the Mortality Information System (*Sistema de Informações sobre Mortalidade*; SIM). SIH-SUS stores information on hospital admissions that occur in the public health-care system, which is the sole provider for approximately 70–80% of the population in Brazil.[1] Data included dates of admission and discharge, primary and secondary diagnosis codes (coded using the International Classification of Diseases, 10th revision – ICD-10). SINAN records information on the notified dengue cases, including the date of symptom onset, symptoms, date and type of laboratory test used for diagnosis. All healthcare facilities in Brazil, private or public, must report dengue cases in the SINAN system. Lastly, information on all deaths occurring in Brazil is registered in SIM, including the death date and underlying cause of death.

The record linkage of the databases was conducted by the Brazilian Ministry of Health using the individual name, their mother's name, birth date, sex (mandatory fields), and city of residence, postal code, and two unique identifiers: their national insurance number (CPF, *Cadastro de Pessoas Físicas*) and national health card number (CNS, *Cartão Nacional de Saúde*) as additional, optional fields.

The linkage process used is known internally as VinculaSUS, which performed matching of the SIHSUS, SINAN and SIM datasets with a centralised and qualified base of national health cards (CADSUS). The matching results provided a new "Patient ID" column on all datasets that is hashed and encrypted to ensure anonymisation. This data workflow is preceded by a preprocessing step consisting of cleaning and standardising textual and numerical fields to ensure a higher probability of success in subsequent matching steps.[2] Records with invalid or null values within the obligatory fields are excluded from the matching process.

As described, VinculaSUS served two main purposes: 1) to identify potential matching records across different databases belonging to the same patient, and 2) to enable data anonymisation by removing identifiable fields and replacing them with a unique, encrypted key field. The complete algorithm has been validated in previous studies[3–6] and was applied here to obtain the source data for this study. The final anonymised dataset was provided to the research team.

We included all individuals admitted to hospitals within the public health system with GBS between January 1, 2023, and December 31, 2024.

Exposure and observation period

The exposure was defined as a laboratory-confirmed dengue infection, i.e. those with a positive NS1 antigen test or a positive dengue RT-PCR test collected within 5 days after

symptom onset, or a positive IgM-based test collected between 6 and 10 days of symptom onset. To account for seasonality, we also included GBS events occurring among individuals without a record of laboratory-confirmed dengue infection during the study period. The observation period for all individuals started on January 1, 2023, and ended on December 31, 2024, or upon death, whichever occurred first.

Outcomes

The primary outcome was hospitalisation for GBS, identified using the ICD-10 code G61.0 in the first three diagnosis fields (primary diagnosis and two secondary codes). The Brazilian guidelines consider GBS diagnosis to be fundamentally clinical, based on rapidly progressive bilateral weakness with hyporeflexia, supported but not dependent on ancillary investigations.[7] The GBS diagnosis and treatment follow the Ministry of Health's Clinical Protocol and Therapeutic Guidelines, which require clinical criteria, with cerebrospinal fluid analysis and electroneuromyography as confirmatory exams. A Brazilian validation study showed that hospitalisations for G61.0 had a positive predictive value of 79% in identifying GBS cases.[8] Similarly, a Taiwanese study showed that G61.0 in the primary, secondary or tertiary position yielded a positive predictive value code of 79%, and when using only the primary position, of 86%.[9]

We also pre-specified several positive and negative control outcomes to assess the likelihood of various sources of bias. The positive control outcomes were acute myocardial infarction (ICD-10 code I21) and stroke (ICD-10 codes I60, I61, I62, I63, and I64), which have been shown to have an increased risk within 15 days of dengue infection.[10] The negative control outcomes were hospitalisation due to fractures of the shoulder and upper arm (ICD-10 code S42), and fractures of the foot (ICD-10 code S92).

Only the first event for each outcome during the study period was included. Individuals with a prior hospitalisation for GBS recorded in the database between 2020 and 2022 were excluded.

Statistical analyses plan

The self-controlled case series (SCCS) design only includes individuals who experienced the event of interest, i.e. GBS hospitalisation.[11] Each individual serves as their own control, thereby eliminating confounding due to time-invariant characteristics. The SCCS estimates the relative incidence of an event is more likely to happen during certain time periods after a person is exposed to something (in this case, a dengue infection) compared to all other observed times within the same person. The method partitions each individual's observation period into exposure risk windows and baseline (unexposed) periods. The SCCS analysis uses conditional Poisson regression to compare the incidence of events across these intervals. Observation time continues after the event occurs because the SCCS addresses a different question than traditional cohort analyses. Rather than estimating the probability of an event, it estimates when, during the observation period, an event occurred, conditional on that event having happened. This type of study relies on four assumptions: 1) Events arise independently within individuals; 2) Occurrence of an event does not influence the

subsequent period of observation; 3) Occurrence of an event does not influence subsequent exposures; 4) Exposures do not influence the ascertainment of events.

The occurrence of GBS may alter the individual's subsequent risk of dengue infection, as GBS may lead to changes in behaviour, such as adopting preventive measures or reducing exposure to mosquito vectors, which could lower the likelihood of dengue infection (violation of assumption 3). We therefore used an extended SCCS approach designed for event-dependent exposures. This method uses a pseudo-likelihood approach in a counterfactual framework to estimate the incidence rate ratio (IRR) and 95% confidence intervals (CIs) by comparing the risk intervals to their corresponding control intervals through maximisation of a Poisson pseudolikelihood. This approach was developed specifically to address bias that might be introduced by event-dependent exposures, as it compares only the rates of events occurring after the exposure.[11] In this model, individuals whose death is attributed to the outcome of interest (GBS) are not censored at the date of death; instead, their observation period continues until the end of the study period. Seasonality was accounted for by adjusting for the month of the year and by including individuals without dengue infection in the model.

We used conditional Poisson regression to compare intra-person incidence rates across different study periods relative to the date of disease onset. We compared the GBS rate within 1–42 days after symptom onset of dengue virus disease with the GBS rate observed during the reference period (from day 43 after symptom onset until Dec 31, 2024). (Figure S1) The 42-day risk period was chosen based on the existing literature on the risk of GBS following infections.[12,13]

We also evaluated an alternative risk period spanning 1 to 84 days, segmented into the following intervals: 1–7, 8–14, 15–28, 29–42, and 43–84 days. The 43-84 day interval was used as a washout period to assess whether the risk returned to baseline. We did not estimate IRRs for any risk interval with two or fewer cases. Day 0 was excluded from the control period.

We conducted multiple sensitivity analyses. First, we repeated the main analysis using only cases confirmed by NS1 or RT-PCR to assess possible misclassification of other arboviruses, such as Zika virus, in the serology testing. Second, we included all dengue cases confirmed by either laboratory or clinical-epidemiological criteria to evaluate potential selection bias arising from only more severe dengue cases being laboratory-confirmed. Finally, we also repeated the main analysis using the standard SCCS approach, including a 28-day pre-exposure period to account for short-term event dependence. In this model, events occurring before the exposure are also considered. (Figure S1)

Effect modification by sex and age (<60 vs ≥60 years) was assessed using chi-square tests to examine heterogeneity between subgroups. In the positive and negative control outcomes, due to large numbers of events, only individuals with dengue infection and a random sample of 5,000 unexposed individuals (i.e., individuals without a recorded dengue infection) were included in the SCCS models. We calculated attributable risk

(AR) using the formula: $\frac{IRR-1}{IRR} * \frac{nr}{N}$.[14] Here, IRR is the incidence rate ratio; nr is the number of GBS hospitalisations in the risk interval; and N is the number of laboratory-confirmed dengue infections.

All secondary analyses (time-stratified risk periods), sensitivity analyses (alternative exposure definitions, standard SCCS model), and subgroup analyses (sex, age) are reported with point estimates and 95% confidence intervals only. The widths of the 95% confidence intervals have not been adjusted for multiplicity and should not be used for hypothesis testing.

The study was approved by the ethics committees of the Instituto Gonçalo Muniz–Oswaldo Cruz Foundation (6.732.188), Salvador, Brazil. Informed consent was waived because the data were deidentified and analysed under strict security procedures, in accordance with the General Data Protection Law (13,709/2018), Article 7, Item IV. All data processing and analyses were performed in R (version 4.1.1), using the tidyverse and SCCS packages.

Supplementary Results

Between 2023 and 2024, a total of 2,357,999 cases of dengue virus disease and 5,055 hospitalisations for GBS were recorded in Brazil. Among the GBS admissions, 147 occurred in individuals with documented dengue infection. (Figures S2 and S3)

Patients admitted for GBS within the predefined risk window, days 1 to 42 after dengue onset, had a lower proportion of males compared with both non-dengue GBS cases and dengue-related GBS admissions occurring outside the risk period. The median hospital stay for GBS during the risk period was similar to that observed among individuals without dengue infection (8 days). However, the group hospitalised during the risk period had a higher proportion of patients requiring ICU (38% versus 22%). Additionally, individuals with dengue infection hospitalised for GBS within the risk period had more cases with dengue with warning signs or severe dengue compared with those whose GBS hospitalisation occurred outside the risk period. (Table S1)

The crude incidence rate ratio (IRR) for developing GBS in the risk period, compared to the control period, was 18.46 (95% CI 12.26 to 27.80). After adjusting for seasonality, the IRR in the model decreased to 17.19 (10.15 to 29.12). In the model that additionally incorporated patients without dengue infection, the IRR was 16.75 (10.97to 25.55, p-value <0.001). The attributable risk (AR) was estimated at 35.49 per million individuals (34.30 to 36.27) (Table 1).

In the analysis stratified by time since dengue infection, the IRR for GBS was highest during the first two weeks, corresponding to more than a 30-fold increase in risk. Although the risk remained significantly elevated in days 15-28, the magnitude was lower (IRR 16.82; 9.86 to 28.70). It declined further to 2.35 (0.82 to 6.75) during days 29-

42 and returned to baseline levels in days 43-84, with an IRR of 0.99 (0.38 to 2.62). (Table 1)

In the subgroup analyses, the incidence of GBS following dengue infection was slightly higher among females (IRR 18.72; 95% CI: 10.12 to 34.62) compared to males (IRR: 15.47; 8.65 to 27.67). IRR were also higher in patients aged ≥ 60 years (26.90; 10.12 to 71.52) than for younger patients (14.54; 9.10 to 23.65). However, none of these differences were statistically significant (p-values for differences in IRR of 0.66 and 0.27, respectively).

The positive control outcome analysis evaluating stroke and AMI, outcomes previously associated with dengue infection, showed IRRs of 2.41 (2.16 to 2.69) and 1.96 (1.72 to 2.23), respectively, during the 1–42-day risk window (Table S3). Week-specific analyses demonstrated a substantial risk in the first week after symptom onset, with an IRR of 6.00 (5.13 to 7.01) for stroke and 3.23 (2.60 to 4.02) for AMI, which remained elevated until 28 days after symptom onset, and returned to baseline levels between days 29 and 42, with IRRs of 1.06 (0.83 to 1.34) for stroke and 1.05 (0.80 to 1.36) for AMI. (Table S4)

The negative control outcomes, hospitalisation for fracture of the shoulder and upper arm, and fracture of the foot, showed no evidence of associations with dengue infection, with IRRs of 0.85 (0.64 to 1.12) and 1.00 (0.68 to 1.46), respectively. (Table S3) Sensitivity analyses restricted to dengue cases confirmed by RT-PCR or NS1 antigen testing showed consistent findings, yielding an IRR of 11.92 (7.33 to 19.38). When dengue cases diagnosed through clinical/epidemiological or any laboratory test were included, the IRR was 17.71 (13.55 to 23.14). (Table 1) Finally, the standard SCCS model, which included a pre-exposure window (days -28 to -1) and applied the same seasonality adjustments while also including individuals without dengue infection, yielded an IRR of 21.92 (15.48 to 31.04).

Supplementary Discussion

Our nationwide study in Brazil provides strong evidence of a markedly increased risk of GBS following acute dengue virus infection. We found that the incidence of GBS was more than 16 times higher during the 42 days after dengue symptoms onset compared to the control period. This risk did not remain constant but followed a distinct temporal pattern, peaking at more than a 30-fold increased risk in the first two weeks after infection and gradually returning to baseline by approximately 43 days post-disease onset.

These findings provide robust epidemiological evidence for the association between dengue infection and GBS. Until now, most of the available evidence had come from small case series and individual case reports.[15–19] One case-control study from Malaysia, including 95 GBS cases, has shown evidence of a higher incidence of dengue infection in GBS cases compared with controls (other neurological diseases).[20] However, this study did not provide a formal measure of association, such as an odds

ratio. Our study strengthens and expands upon these earlier clinical observations on a national scale and contributes novel quantified risk estimates by time since infection.

The magnitude of this observed risk is comparable to that reported for other infectious triggers of GBS. In previous SCCS studies evaluating the risk of GBS after influenza, a study conducted in England found an IRR of 16 between the 0-30-day period after disease onset,[21] while a study from Italy assessing gastrointestinal infections found an IRR of 41 during the 0-42-day window.[22] A systematic review of case-control studies assessing the risk following *Campylobacter jejuni* infection reported odds ratios ranging from 3 to 41.[12] *Campylobacter jejuni* infection is widely regarded as the most common infectious trigger for GBS globally, accounting for an estimated 30% of GBS cases worldwide.[23] However, in tropical areas such as Brazil and India, arboviral infections appear more prevalent among GBS cases than *Campylobacter jejuni*.[19,24,25] Studies assessing the relationship between the Zika virus and GBS have reported a high proportion of GBS cases with laboratory evidence of recent Zika virus infection; during the 2016 outbreak, a study reported that up to 97% of GBS cases had confirmed prior Zika virus infection.[26] It has been estimated that 200 GBS cases may occur per million of Zika virus infections.[27] In contrast, our study estimated an excess risk of 30 GBS cases per million dengue virus infections. Although the individual risk of GBS following dengue infection appears lower than that following Zika infection, the overall public health impact of dengue is much greater due to its substantially higher incidence. In 2024, an estimated 14 million dengue cases occurred compared with 45,000 Zika cases.[28,29] Based on these figures, dengue infection would account for approximately 336 to 518 GBS cases, whereas Zika would account for only about nine cases.

Our subgroup analyses did not find statistically significant evidence that the association between dengue and GBS differs by sex or age group. Nonetheless, the point estimates for the relative incidence were higher among individuals aged 60 years or older. The lack of statistical significance is likely attributable to the limited number of GBS cases within each stratum, which reduces the statistical power to detect true subgroup differences. Even so, the observed point estimates support biologically plausible hypotheses. The elevated incidence rate ratio in older adults (≥60 years) may reflect age-related alterations in immune response, such as immunosenescence or chronic, progressive low-grade inflammation associated with aging ('inflamm-aging'), which can contribute to dysregulated immune activation following infection.[30]

Molecular mimicry in Guillain–Barré syndrome is typically expected to manifest 15–42 days after infection.[31] However, we observed an elevated incidence rate ratio (IRR) within the first 7 days. This unusually rapid onset is consistent with case series reporting short intervals between dengue or Zika symptom onset and neurological manifestations (median 6–7 days).[19,32] In contrast, Guillain–Barré syndrome following *Campylobacter jejuni* infection or influenza generally exhibits a longer latency, with neurological symptoms appearing approximately 10 days after infection.[21,33] Precise viral–host molecular targets are still not fully characterised, and mechanisms may differ between viruses and GBS subtypes.

This study has several strengths, including the use of comprehensive national healthcare data, laboratory-confirmed dengue cases, and the application of SCCS methodology, which minimises time-invariant confounding. We also applied a modified SCCS design specific to situations with event-dependent exposure, as the occurrence of a debilitating event like GBS could alter an individual's subsequent behaviour, potentially reducing their exposure to mosquito bites and thus their risk of a future dengue infection. In line with methodological guidance, we also included individuals without the exposure (dengue infection).[11] Our sensitivity analysis, restricted to dengue cases confirmed by RT-PCR or NS1 antigen test, which have high specificity and are less prone to cross-reactivity with other flaviviruses like Zika virus, showed a consistently strong association (IRR 11.92). The attenuation of this estimate compared to the primary analysis (IRR 16.75) is plausibly explained, at least in part, by the misclassification of some Zika-induced GBS as dengue-associated GBS in the main cohort, given the known IgM serological cross-reactivity and the co-circulation of both arboviruses in Brazil.[34] The analysis, including dengue cases without laboratory confirmation, produced a slightly higher IRR (17.7). Although this broader case definition increases the potential for exposure misclassification, particularly from other undifferentiated febrile illnesses, its consistency with our primary analysis is reassuring. These findings suggest that our results are unlikely to be explained solely by selection bias, such as the preferential identification of more severe dengue cases that are more likely to undergo laboratory testing.

Our negative and positive control outcomes also support the robustness of our findings. The positive control outcomes of stroke and AMI presented smaller effects than those found from a Taiwanese study also employing SCCS.[16] One possible reason for the difference is that our study used the extended SCCS, while the Taiwanese study used the standard SCCS. Because individuals with prior stroke or AMI are likely to modify behaviours that influence their probability of subsequent exposure, the use of the standard SCCS in that study may have biased the estimates upward, as seen in our analysis employing the standard SCCS with a higher IRR than the model using the extended SCCS. We also found a longer period of increased risk, extending up to 28 days; this temporal pattern is similar to that reported for chikungunya disease.[35] However, some limitations must be acknowledged. We were unable to distinguish between DENV serotypes and primary or secondary dengue, and we also couldn't distinguish between the clinical subtypes of GBS (e.g., AIDP vs. AMAN). This information would provide deeper mechanistic insights. Additionally, we were also unable to assess the occurrence of GBS hospitalisations in the private healthcare sector; however, dengue infection is strongly associated with socioeconomic vulnerability,[36] being more common in vulnerable populations, which are usually not covered by private insurance, making it unlikely that a substantial number of dengue-associated GBS would have been missed.

Our findings have significant clinical and epidemiological implications and can inform efforts to mitigate the burden of neglected tropical diseases in affected populations, in line with the Sustainable Development Goals 2030 Agenda. For clinicians, these findings underscore the need for a suspicion of GBS in patients presenting with neurological symptoms during or shortly after a dengue infection. Early recognition and

diagnosis of GBS are crucial, as the timely administration of immunotherapy, either intravenous immunoglobulin or plasmapheresis, is known to halt the disease's progression and significantly improve long-term outcomes.[37] From a public health perspective, these results quantify a substantial, and likely under-recognised, neurological burden attributable to dengue. Although GBS is a rare disease, the enormous scale of dengue epidemics in Brazil and elsewhere transforms this rare outcome into a significant public health problem. During the two-year study period alone, over 2.3 million laboratory-confirmed dengue cases were notified in Brazil, and in 2024, the country registered over 7 million suspected cases. Given that GBS events triggered by dengue are, in principle, vaccine-preventable, our findings substantially strengthen the rationale for robust primary prevention, including intensified vector control and the strategic expansion of dengue vaccination programs.

Author Contributions

Author contributions are provided according to Contributor Roles Taxonomy (CRediT):
Conceptualization: TCS
Data curation: NMS, MCA
Formal analysis: TCS
Funding acquisition: TCS, MB-N, VSB
Investigation: TCS, MB-N, VSB, GVA
Methodology: TCS, NP
Project administration: TCS, MB-N, VSB
Resources: MB-N, NMS, LXST, GVA
Software: TCS
Supervision: MB-N, VSB, NP
Visualization: TCS, NP
Writing – original draft: TCS
Writing – review and editing: MB-N, VSB, GVA, NP, EP

Competing Interests

The authors declare no conflicts of interest. The funders of the study did not have any further role in study design, data collection, data analysis, data interpretation, or writing of the report.

Acknowledgements

The authors acknowledge DATASUS for its diligent work in providing the unidentified Brazilian databases

Supplementary Figures

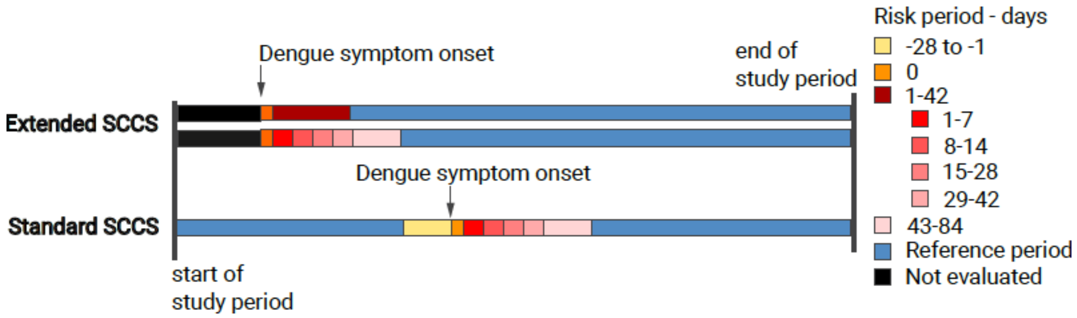

Figure S1: Illustration of the risk period for the extended and standard self-controlled case cases. In the extended SCCS, exposures after an event are disregarded and considered as missing.

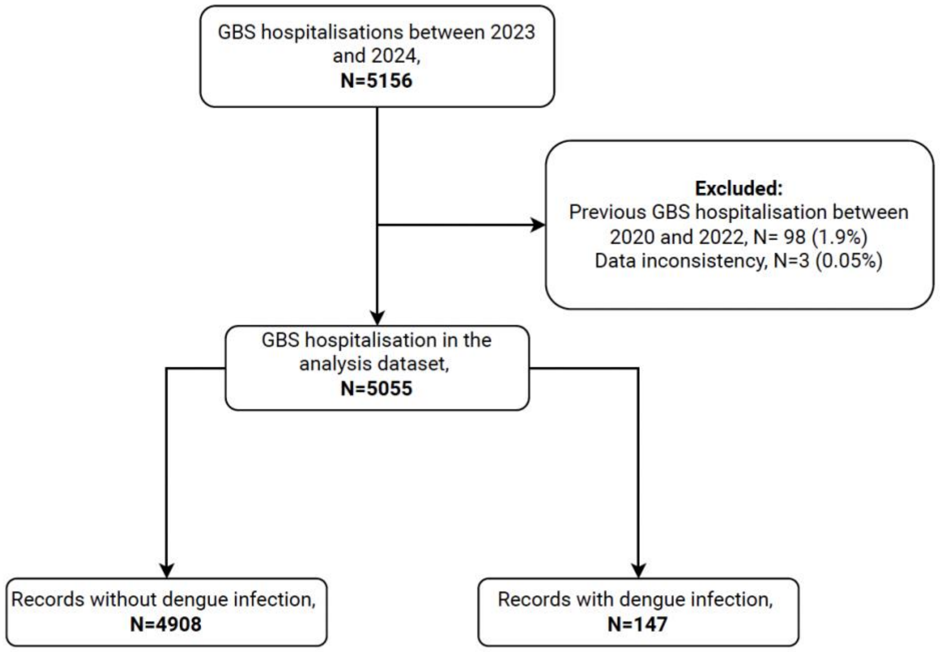

Figure S2: Selection of study participants. GBS: Guillain-Barré syndrome. Data inconsistency = individuals with a date of death before the date of hospitalisation.

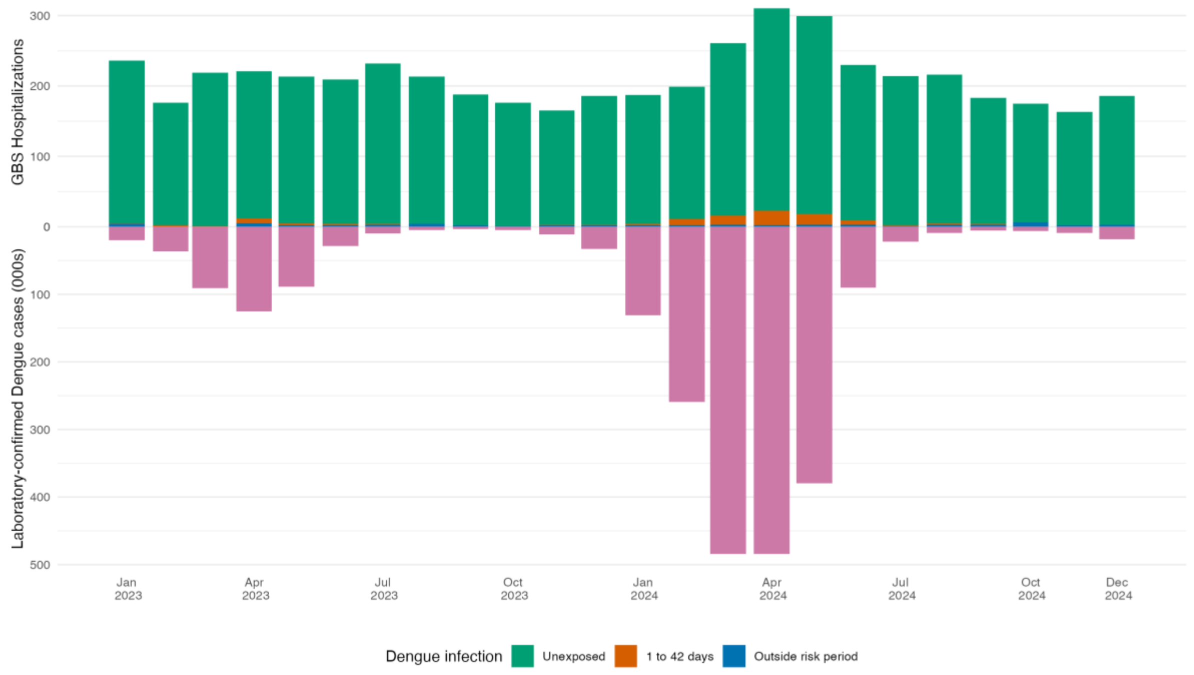

Figure S3: Number of Guillain-Barré syndrome (GBS) hospitalisations and laboratory-confirmed dengue cases (in thousands) by month during the study period. GBS cases are stratified by dengue infection status, unexposed (uninfected), 1 to 42 days (GBS hospitalisation 1 to 42 days after dengue symptom onset) and outside risk period (GBS hospitalisation before dengue symptom onset or after 43 days of symptom onset)

Supplementary Tables

Table S1: Baseline Characteristics of Patients Who had a GBS hospitalisation within the study Period.

			Dengue cases	
**Characteristic**	**No dengue infection**	**Pre risk period (≤0 day)**	**Risk period (1-42 days)**	**Post risk period (≥43 days)**
	**N = 4,908**	**N = 26**	**N = 89**	**N = 32**
**Sex-Male**	2,775 (57%)	15 (58%)	43 (48%)	17 (53%)
**Age years, median (IQR)**	44 (25, 60)	38 (16, 60)	49 (33, 62)	41 (27, 56)

**Age (≥60 years)**	1,251 (25%)	7 (27%)	26 (29%)	6 (19%)
**Race/ethnicity**				
Asian	80 (1.6%)	2 (7.7%)	3 (3.4%)	0 (0%)
White	1,878 (39%)	14 (54%)	48 (54%)	16 (50%)
Indigenous	14 (0.3%)	1 (3.8%)	1 (1.1%)	0 (0%)
Mixed (Pardo)	2,693 (55%)	9 (35%)	31 (35%)	16 (50%)
Black	200 (4.1%)	0 (0%)	6 (6.7%)	0 (0%)
Missing	43	0	0	0
**Length of stay (days), median (IQR)**	8 (4, 14)	6 (4, 15)	8 (6, 14)	8 (6, 14)
**ICU during hospitalisation**	1,099 (22%)	6 (23%)	34 (38%)	10 (31%)
**In-hospital death**	216 (4.4%)	0 (0%)	4 (4.5%)	2 (6.3%)
**Diagnosis GBS -hospitalisation**				
Main cause	4,764 (97%)	26 (100%)	80 (90%)	29 (91%)
Secondary diagnosis	144 (2.9%)	0 (0%)	9 (10%)	3 (9.4%)
**Region**				
North	413 (8.4%)	0 (0%)	1 (1.1%)	0 (0%)
Northeast	1,101 (22%)	2 (7.7%)	6 (6.7%)	3 (9.4%)
Southeast	1,939 (40%)	12 (46%)	45 (51%)	16 (50%)
South	960 (20%)	7 (27%)	23 (26%)	9 (28%)
Central-West	495 (10%)	5 (19%)	14 (16%)	4 (13%)
**Type of test**				
NS1/RT-PCR	0 (NA%)	17 (65%)	54 (61%)	25 (78%)
Serology (IgM)	0 (NA%)	9 (35%)*	35 (39%)†	7 (22%)‡
**Dengue classification**				

Dengue without alarm signs	25 (96%)	74 (83%)	30 (94%)
Dengue with alarm signs	0 (0%)	7 (7.9%)	1 (3.1%)
Severe dengue	1 (3.8%)	8 (9.0%)	1 (3.1%)

**Only tested with serological tests*
† 30 out of 35 were tested only with serological tests, 5 tested negative in the PCR/NS1 (between 6 and 9 days after symptom onset) and positive in IgM.
‡ 6 out of 7 were tested only with serological tests, 1 tested negative in the PCR/NS1 (8 days after symptom onset) and positive in IgM.

Table S2. Baseline characteristics of patients who tested positive for dengue and who had an outcome of interest within the study period. Only for Guillain-Barré syndrome (GBS) clinical cases are also included.

Variable	Stroke	AMI	Foot fracture	Shoulder and Upper Arm fracture	GBS-Clinical*
	N = 3,660	N = 3,072	N = 554	N = 1,207	N = 408
**Sex-Male**	1,802 (49%)	1,859 (61%)	401 (72%)	742 (61%)	208 (51%)
**Age years, median (IQR)**	66 (55, 75)	63 (54, 71)	34 (23, 48)	35 (19, 56)	44 (25, 60)
**Race/ethnicity**					
Asian	36 (1.0%)	38 (1.2%)	10 (1.8%)	14 (1.2%)	12 (3.0%)
White	2,129 (58%)	1,820 (60%)	241 (44%)	618 (51%)	198 (49%)
Indigenous	3 (<0.1%)	3 (<0.1%)	1 (0.2%)	6 (0.5%)	3 (0.7%)
Mixed (Pardo)	1,289 (35%)	1,056 (35%)	281 (51%)	527 (44%)	173 (43%)
Black	185 (5.1%)	138 (4.5%)	20 (3.6%)	41 (3.4%)	20 (4.9%)
Missing	18	17	1	1	2
**Length of stay (days), median (IQR)**	4 (2, 8)	4 (2, 8)	2.0 (1.0, 4.0)	2.0 (1.0, 4.0)	8 (5, 14)
**ICU during hospitalisation**	770 (21%)	1,267 (41%)	2 (0.4%)	28 (2.3%)	116 (28%)
**In-hospital death**	334 (9.1%)	147 (4.8%)	0 (0%)	1 (<0.1%)	18 (4.4%)
**Region**					
North	44 (1.2%)	19 (0.6%)	12 (2.2%)	22 (1.8%)	9 (2.2%)

Northeast	127 (3.5%)	75 (2.4%)	32 (5.8%)	79 (6.5%)	45 (11%)
Southeast	2,202 (60%)	1,948 (63%)	342 (62%)	722 (60%)	196 (48%)
South	1,023 (28%)	737 (24%)	121 (22%)	275 (23%)	99 (24%)
Central-West	264 (7.2%)	293 (9.5%)	47 (8.5%)	109 (9.0%)	59 (14%)
**Type of test**					
NS1/RT-PCR	2,875 (79%)	2,472 (80%)	445 (80%)	985 (82%)	96 (24%)
Serology (IgM)	785 (21%)	600 (20%)	109 (20%)	222 (18%)	51 (12%)
**Dengue classification**					
Dengue without alarm sign	3,384 (92.5%)	2,922 (95%)	545 (98.4%)	1,175 (97.4%)	361 (88%)
Dengue with alarm signs	201 (5.5%)	107 (3.5%)	9 (1.6%)	28 (2.3%)	23 (5.6%)
Severe dengue	73 (2.0%)	43 (1.4%)	0	4 (0.3%)	24 (5.9%)

***Including those who also had dengue infection confirmed through clinical or laboratory criteria**

Table S3. Incidence Rate Ratios after Laboratory Confirmed Dengue Infection in the risk period of 1 to 42 days post symptom onset in the positive and negative control outcomes.

Analysis	Number of events	Incidence rate ratio (95% CI)	Attributable risk per million of infections
Acute myocardial infarction	356	1.96 (1.72 to 2.23)	73.95 (63.2 to 83.27)
Stroke	594	2.41 (2.16 to 2.69)	147.38 (135.28 to 158.26)
Should and upper arm fracture	59	0.85 (0.64 to 1.12)	-4.42 (-14.07 to 2.68)
Foot fracture	31	1.00 (0.68 to 1.46)	0 (-6.19 to 4.14)

Table S4. Incidence Rate Ratios (IRR) of Stroke and Acute Myocardial Infarction (AMI) following Laboratory Confirmed Dengue Infection in the risk period of 1 to 84 days post symptom onset, stratified by time.

	Stroke		AMI	
**Term**	**Number of events**	**IRR (95% CI)**	**Number of events**	**IRR (95% CI)**
Days 1-7	254	6.00 (5.13 to 7.01)	110	3.23 (2.60 to 4.02)
Days 8-14	126	3.32 (2.74 to 4.03)	79	2.66 (2.10 to 3.37)
Days 15-28	141	1.98 (1.66 to 2.37)	107	1.84 (1.49 to 2.26)
Days 29-42	73	1.06 (0.83 to 1.34)	60	1.05 (0.80 to 1.36)
Days 43-84	236	1.14 (0.99 to 1.32)	163	0.95 (0.80 to 1.13)

Supplementary References:

1. Cerqueira-Silva T, Barral-Netto M, Boaventura VS. Effect of Brazil's national human papillomavirus vaccination programme on the incidence of cervical cancer and cervical intraepithelial neoplasia grade 3 in women aged 20–24 years: a population-based study. The Lancet Global Health 2025;13(10):e1715–22.

2. Guerra Junior AA, Pereira RG, Gurgel EI, et al. Building the National Database of Health Centred on the Individual: Administrative and Epidemiological Record Linkage - Brazil, 2000-2015. Int J Popul Data Sci 2018;3(1):446.

3. Cerqueira-Silva T, Katikireddi SV, de Araujo Oliveira V, et al. Vaccine effectiveness of heterologous CoronaVac plus BNT162b2 in Brazil. Nat Med 2022;28(4):838–43.

4. Cerqueira-Silva T, Andrews JR, Boaventura VS, et al. Effectiveness of CoronaVac, ChAdOx1 nCoV-19, BNT162b2, and Ad26.COV2.S among individuals with previous SARS-CoV-2 infection in Brazil: a test-negative, case-control study. The Lancet Infectious Diseases 2022;22(6):791–801.

5. Katikireddi SV, Cerqueira-Silva T, Vasileiou E, et al. Two-dose ChAdOx1 nCoV-19 vaccine protection against COVID-19 hospital admissions and deaths over time: a retrospective, population-based cohort study in Scotland and Brazil. The Lancet [Internet] 2021 [cited 2021 Dec 21];399(10319). Available from: https://www.thelancet.com/journals/lancet/article/PIIS0140-6736(21)02754-9/fulltext

6. Cerqueira-Silva T, Shah SA, Robertson C, et al. Effectiveness of mRNA boosters after homologous primary series with BNT162b2 or ChAdOx1 against symptomatic infection and severe COVID-19 in Brazil and Scotland: A test-negative design case–control study. PLoS Med 2023;20(1):e1004156.

7. Brasil M da S. Protocolo Clínico e Diretrizes Terapêuticas da Síndrome de Guillain-Barré. 2021;

8. Oliveira AF de M, Gallo LG, Bastos MM, et al. Sensitivity of Guillain-Barre Syndrome Surveillance in the Brazilian Federal District, using the Capture-Recapture Method. The Journal of Infection in Developing Countries 2021;15(10):1507–14.

9. Hsieh C-Y, Chen P-T, Shao S-C, Lin S-J, Liao S-C, Lai EC-C. Validating ICD-10 Diagnosis Codes for Guillain-Barré Syndrome in Taiwan’s National Health Insurance Claims Database. CLEP 2024;16:733–42.

10. Wei K-C, Sy C-L, Wang W-H, Wu C-L, Chang S-H, Huang Y-T. Major acute cardiovascular events after dengue infection–A population-based observational study. PLOS Neglected Tropical Diseases 2022;16(2):e0010134.

11. Ghebremichael-Weldeselassie Y, Jabagi MJ, Botton J, et al. A modified self-controlled case series method for event-dependent exposures and high event-related mortality, with application to COVID-19 vaccine safety. Statistics in Medicine 2022;41(10):1735–50.

12. Wachira VK, Peixoto HM, de Oliveira MRF. Systematic review of factors associated with the development of Guillain–Barré syndrome 2007–2017: what has changed? Tropical Medicine & International Health 2019;24(2):132–42.

13. Grave C, Boucheron P, Rudant J, et al. Seasonal influenza vaccine and Guillain-Barré syndrome: A self-controlled case series study. Neurology 2020;94(20):e2168–79.

14. Xu S, Sy LS, Hong V, et al. Ischemic Stroke After Bivalent COVID-19 Vaccination: Self-Controlled Case Series Study. JMIR Public Health and Surveillance 2024;10(1):e53807.

15. Sanchez-Landers M, Rodriguez-Benites AF. Síndrome de Guillain-Barre asociado a dengue previo: reporte de caso. Rev Peru Med Exp Salud Publica 2025;88–91.

16. Imtiaz H, Khan AF, Khan S. Dengue-induced Guillain–Barre syndrome: a case series. The Egyptian Journal of Neurology, Psychiatry and Neurosurgery 2023;59(1):149.

17. Fragoso YD, Gomes S, Brooks JBB, et al. Guillain-Barré syndrome and dengue fever: report on ten new cases in Brazil. Arq Neuro-Psiquiatr 2016;74:1039–40.

18. Hassan IN, Yaqub S, Ibrahim M, Aljaili G, Abuassa N. A rare neurological complication of dengue: Guillain-Barré Syndrome in a dengue fever patient. IDCases 2025;39:e02160.

19. Effiong MG, Yakubu AO, Lawal FI, Ojoh UH, Adeniyi TO. Dengue virus infection and Guillain–Barré syndrome: a systematic review of clinical characteristics, outcomes, and predictors of severity. BMC Infect Dis [Internet] 2025 [cited 2025 Dec 30];Available from: https://doi.org/10.1186/s12879-025-12428-7

20. Tan C-Y, Razali SNO, Goh KJ, Sam I-C, Shahrizaila N. Association of dengue infection and Guillain-Barré syndrome in Malaysia. J Neurol Neurosurg Psychiatry 2019;90(11):1298–300.

21. Stowe J, Andrews N, Wise L, Miller E. Investigation of the Temporal Association of Guillain-Barré Syndrome With Influenza Vaccine and Influenzalike Illness Using the United Kingdom General Practice Research Database. Am J Epidemiol 2009;169(3):382–8.

22. Galeotti F, Massari M, D'Alessandro R, et al. Risk of Guillain-Barré syndrome after 2010–2011 influenza vaccination. Eur J Epidemiol 2013;28(5):433–44.

23. Finsterer J. Triggers of Guillain–Barré Syndrome: Campylobacter jejuni Predominates. Int J Mol Sci 2022;23(22):14222.

24. Dutta D, Debnath M, Nagappa M, et al. Antecedent infections in Guillain-Barré syndrome patients from south India. Journal of the Peripheral Nervous System 2021;26(3):298–306.

25. Vieira MA da C e S, Costa CHN, Linhares A da C, et al. Potential role of dengue virus, chikungunya virus and Zika virus in neurological diseases. Mem Inst Oswaldo Cruz 2018;113:e170538.

26. Parra B, Lizarazo J, Jiménez-Arango JA, et al. Guillain–Barré Syndrome Associated with Zika Virus Infection in Colombia. New England Journal of Medicine 2016;375(16):1513–23.

27. Mier-Y-Teran-Romero L, Delorey MJ, Sejvar JJ, Johansson MA. Guillain-Barré syndrome risk among individuals infected with Zika virus: a multi-country assessment. BMC Med 2018;16(1):67.

28. Zika: Analysis by country - PAHO/WHO | Pan American Health Organization [Internet]. [cited 2025 Nov 2];Available from: https://www.paho.org/en/arbo-portal/zika-data-and-analysis/zika-analysis-country

29. Haider N, Hasan MN, Onyango J, et al. Global dengue epidemic worsens with record 14 million cases and 9000 deaths reported in 2024. International Journal of Infectious Diseases 2025;158:107940.

30. Li X, Li C, Zhang W, Wang Y, Qian P, Huang H. Inflammation and aging: signaling pathways and intervention therapies. Sig Transduct Target Ther 2023;8(1):239.

31. Leonhard SE, Papri N, Querol L, Rinaldi S, Shahrizaila N, Jacobs BC. Guillain–Barré syndrome. Nat Rev Dis Primers 2024;10(1):97.

32. Cao-Lormeau V-M, Blake A, Mons S, et al. Guillain-Barré Syndrome outbreak associated with Zika virus infection in French Polynesia: a case-control study. The Lancet 2016;387(10027):1531–9.

33. Rees JH, Soudain SE, Gregson NA, Hughes RAC. Campylobacter jejuni Infection and Guillain–Barré Syndrome. New England Journal of Medicine 1995;333(21):1374–9.

34. Zaidi MB, Cedillo-Barron L, González y Almeida ME, et al. Serological tests reveal significant cross-reactive human antibody responses to Zika and Dengue viruses in the Mexican population. Acta Tropica 2020;201:105201.

35. Cerqueira-Silva T, Pescarini JM, Cardim LL, et al. Risk of death following chikungunya virus disease in the 100 Million Brazilian Cohort, 2015–18: a matched cohort study and self-controlled case series. The Lancet Infectious Diseases 2024;24(5):504–13.

36. Sansone NMS, Boschiero MN, Marson FAL. Dengue outbreaks in Brazil and Latin America: the new and continuing challenges. International Journal of Infectious Diseases 2024;147:107192.

37. Shahrizaila N, Lehmann HC, Kuwabara S. Guillain-Barré syndrome. The Lancet 2021;397(10280):1214–28.
